# Associations between androgen levels and endurance training‐induced changes in body composition and physical performance in premenopausal females

**DOI:** 10.14814/phy2.70857

**Published:** 2026-04-14

**Authors:** Vera M. Salmi, Jari E. Karppinen, Maarit Lehti, Heikki Kyröläinen, Johanna K. Ihalainen, Ritva S. Mikkonen

**Affiliations:** ^1^ Faculty of Sport and Health Sciences University of Jyväskylä Jyväskylä Finland; ^2^ Obesity Research Unit, Research Program for Clinical and Molecular Metabolism University of Helsinki Helsinki Finland; ^3^ Finnish Institute of High Performance Sport KIHU Jyväskylä Finland; ^4^ Sports Technology Unit, Faculty of Sport and Health Sciences University of Jyväskylä Vuokatti Finland

**Keywords:** androgens, combined oral contraceptives, DHEA‐S, eumenorrheic, female physiology, testosterone

## Abstract

We investigated whether a moderate‐intensity continuous training (MICT) for two menstrual/contraceptive cycles (~8 weeks) alters endogenous androgen concentrations in premenopausal females, and whether baseline androgen concentrations or their changes from baseline to post‐intervention are associated with adaptations in body composition and physical performance. Serum total/free testosterone, dihydrotestosterone, androstenedione, dehydroepiandrosterone (DHEA), DHEA‐sulfate (DHEA‐S), and sex hormone‐binding globulin were analyzed in follicular/luteal phases in eumenorrheic females (EUM, *n* = 18) and in inactive/active phases in combined oral contraceptive users (COC) (*n* = 8). Fat‐free mass (FFM), fat mass, counter movement jump (CMJ), maximal isometric force (*F*_max_), and aerobic capacity (V̇O_2peak_) were measured across MICT. Androgen concentrations remained stable from baseline to post‐intervention. Body composition remained statistically unchanged, while V̇O_2peak_ increased by 0.05 L·min^−1^ from baseline to post‐intervention at luteal/active phase (standardized estimate *β* = 0.16, *p* = 0.046). Baseline DHEA‐S was positively associated with changes (∆) in FFM‐adjusted V̇O_2peak_ (*β* = 0.31, *p* = 0.026), and ∆DHEA‐S was negatively associated with ∆V̇O_2peak_ at follicular/inactive phase (*β* = −0.55, *p* = 0.030). Baseline total testosterone (*β* = −1.86, *p* = 0.042) and DHEA‐S (*β* = −0.36, *p* = 0.016) were negatively associated with ∆CMJ at luteal/active phase. These associations were no longer significant after adjusting for FFM and fat mass. Although baseline androgen levels and their changes were not consistently related to body composition or physical performance changes, DHEA‐S concentrations may be associated with endurance training–related changes in V̇O_2peak_.

## 1 BACKGROUND

Endurance training induces both neuromuscular and cardiovascular adaptations: it increases blood and red cell volume (Convertino, 1991), cardiac output and stroke volume (Hellsten & Nyberg, 2016), maximal oxygen consumption, capillary density (Andersen & Henriksson, 1977), and glycogen storage, while also enhancing mitochondrial biogenesis, fat utilization, and processing of lactate (Holloszy & Coyle, 1984). These changes in the central and peripheral tissues ultimately lead to enhanced endurance performance. In addition, endurance training can aid in reducing body weight and fat mass (FM) in premenopausal females (Behboudi & Eizadi, 2017; Smith et al., 2011). This can positively influence aerobic capacity through reduced energy cost on movement, especially in weight‐bearing activities such as running, as moving a greater mass consumes more oxygen and can affect the economy of human locomotion (Pate et al., 1987).

Moderate‐intensity continuous training (MICT), usually performed at 60%–70% of maximum aerobic capacity, is a widely used method to support modification of body composition and to improve physical performance in the general population. Improvements in peak (V̇O_2peak_) or maximal oxygen uptake (V̇O_2max_) after regular (3–4 sessions weekly), continuous (30–45 min), moderate‐to‐vigorous‐intensity [60%–80% of V̇O_2peak_, 60%–75% of maximal heart rate (HR_max_)] exercise program (lasting from 5 weeks to 3 months) are well documented in literature in premenopausal females (Behboudi & Eizadi, 2017; Kong et al., 2016). Although shorter interventions (7 weeks, 5 day: 2 day exercise: rest protocol, 60 min at an intensity of 60% of V̇O_2peak_) may not induce changes in fat‐free mass (FFM) and FM (Carter et al., 2001), longer interventions (~16 weeks, 5 times a week for 30 min at an intensity of 65% of the age‐predicted HR_max_ gradually increased to 85%) can increase lean mass in previously sedentary eumenorrheic females (Smith et al., 2011). However, the effect of endurance‐only training on maximal strength and power performance is not well investigated, especially in females.

In females, androgens are secreted by both ovaries and adrenal glands. The main androgens secreted by the ovaries are testosterone and dihydrotestosterone (DHT) (Longcope, 1986). Androgen precursors androstenedione, dehydroepiandrosterone (DHEA) and its sulfate (DHEA‐S) are secreted from the ovaries and adrenal glands and can be converted to testosterone and DHT in the ovary or through peripheral conversion in tissues (Burger, 2002; Longcope, 1986). Testosterone and DHT circulate in the bloodstream mainly bound to sex hormone‐binding globulin (SHBG) (Burger, 2002; Fiers et al., 2018), while the free hormone fraction can bind to the androgen receptor exerting effects on target tissues. Cross‐sectional studies have found that endogenous androgen concentrations are positively associated with lean mass and physical performance in female athletes (Eklund et al., 2017), while DHEA‐S concentrations are negatively associated with V̇O_2peak_ in recreationally active and trained premenopausal females (Salmi et al., 2025). In premenopausal females, free androgen index, but not total testosterone, has been positively associated with lean mass index (Alexander et al., 2021) and with resistance training‐induced changes in muscle mass and strength in untrained normally menstruating females and females using HC (Alexander et al., 2024), suggesting that androgen concentrations may interact with training adaptations in body composition and physical performance.

Concentrations of androgens appear to fluctuate during the menstrual cycle. Previous research, including analyses from our laboratory, shows that the ovulatory peak of estradiol is accompanied by a peak in concentrations of total testosterone in healthy, premenopausal females (Rothman et al., 2011; Salmi et al., 2026). This cyclical variation and endogenous production of sex steroids can be suppressed with the use of combined oral contraceptives (COC), which act by negative feedback on the hypothalamic–pituitary–ovarian axis. This results in significantly lower serum concentrations of estradiol, progesterone (Mishell et al., 1972; Salmi et al., 2026) and androgens (Salmi et al., 2026; Thorneycroft et al., 1999; van der Vange et al., 1990; Wiegratz et al., 2003), and relatively stable androgen concentrations between the pill‐taking phase i.e., active phase and pill‐free phase i.e., inactive phase (Salmi et al., 2026). In females using COC, the availability of free androgens can also be altered as COCs increase concentrations of SHBG (Thorneycroft et al., 1999; van der Vange et al., 1990; Wiegratz et al., 2003).

Some sex hormone concentrations can be altered when examining the overall effect of strength and endurance exercise training (Shahid et al., 2024). Keizer et al. (1987) found decreases in basal estradiol and total testosterone concentrations in the luteal phase, and in DHEA‐S concentrations in both follicular and luteal phases, after a 3‐month endurance training program in untrained eumenorrheic females. The training consisted of running and cycling, the training volume and intensity progressively increasing from approximately 60% of V̇O_2max_ to 70%–80% of V̇O_2max_. On the other hand, some research has reported no significant change in basal testosterone concentrations during an approximately 16‐week aerobic exercise program in healthy, sedentary, eumenorrheic females (Smith et al., 2011), during an approximately 13.5‐month progressive running program in healthy, regularly menstruating females (Boyden et al., 1983), and during an 18–20‐month running program in previously untrained females (Keizer et al., 1989), even with concurrent decreases in estradiol concentrations and changes towards leaner body composition (Boyden et al., 1983). Furthermore, physiological concentrations of exogenous testosterone have been shown to increase aerobic running time and lean mass (Hirschberg et al., 2020).

Variability in the results might be related to factors such as training mode, intervention duration, measurement schedules, and participant characteristics. It is largely unknown how endogenous basal androgen concentrations contribute to training adaptations, particularly in endurance training in eumenorrheic females and in females using COCs. Furthermore, the influence of training‐induced changes in basal androgen concentrations on training adaptations is not well understood. Therefore, our aim was to investigate (1) whether endogenous androgen concentrations change in response to a MICT intervention equivalent to the length of participants' two menstrual/contraceptive cycles (~8 weeks), and (2) whether baseline androgen concentrations or their potential changes from baseline to post‐intervention are associated with endurance training‐induced changes in body composition and physical performance. We also explored whether these associations differ depending on the menstrual cycle/COC phase in healthy, recreationally active premenopausal females.

## 2 METHODS

### 2.1 Participants

This study uses data from “the women's menstrual cycle and endurance training (NaisQs) study” conducted in the University of Jyväskylä, Finland. Participants were recruited via advertisements in sport halls, gyms, and public places as well as the University of Jyväskylä's mailing lists, website, and social media channels. A total of 190 females expressed their interest to the study by filling the registration form online. Participants were excluded if they did not meet the following inclusion criteria: healthy, aged 18–35 years, a self‐reported regular 26–35‐day menstrual cycle or use of combined monophasic oral contraceptives for at least 3 months prior to the study and self‐reported body mass index (BMI) 19.5–35 kg·m^−2^. Most of the participants were recreationally active (Tier 1), defined according to the Participant Classification Framework (McKay et al., 2021), meaning that they participated in a variety of sports with no engagement in goal‐oriented training. Some of the participants were closer to Tier 0 (McKay et al., 2021), but as none of them were completely sedentary, the term recreationally active is used. Participants were excluded if they smoked, had a chronic disease, reported taking pharmacological agents known to affect metabolic or exercise‐related responses (with the exception of COCs). These agents included antidepressants (such as selective serotonin reuptake inhibitors and serotonin‐norepinephrine reuptake inhibitors), thyroid hormone preparations, antirheumatic drugs, and central nervous system stimulants.

Further exclusion criteria were amenorrhea, clinically diagnosed polycystic ovary syndrome (PCOS) or any other disease or condition that could affect ovarian function and physical disabilities or musculoskeletal or endocrine disorders affecting metabolism, running ability or physical performance. Participants volunteering for the study and fulfilling the inclusion criteria completed a health questionnaire that was screened and approved by a medical doctor prior to enrollment. Participants were recruited into two groups: females not using any hormonal contraception at least 1 year prior to the study with a self‐reported normal menstrual cycle length of 26–35 days and females using monophasic COC pills for at least 3 months prior to the study. The study followed the principles of the Declaration of Helsinki, and the Ethical Committee of the University of Jyväskylä approved the methodology of NaisQs study (1519/13.00.04/2021). All participants received detailed information about the design, measurements, procedures, and possible risks of the study and signed a written informed consent before the study onset.

**FIGURE 1:**
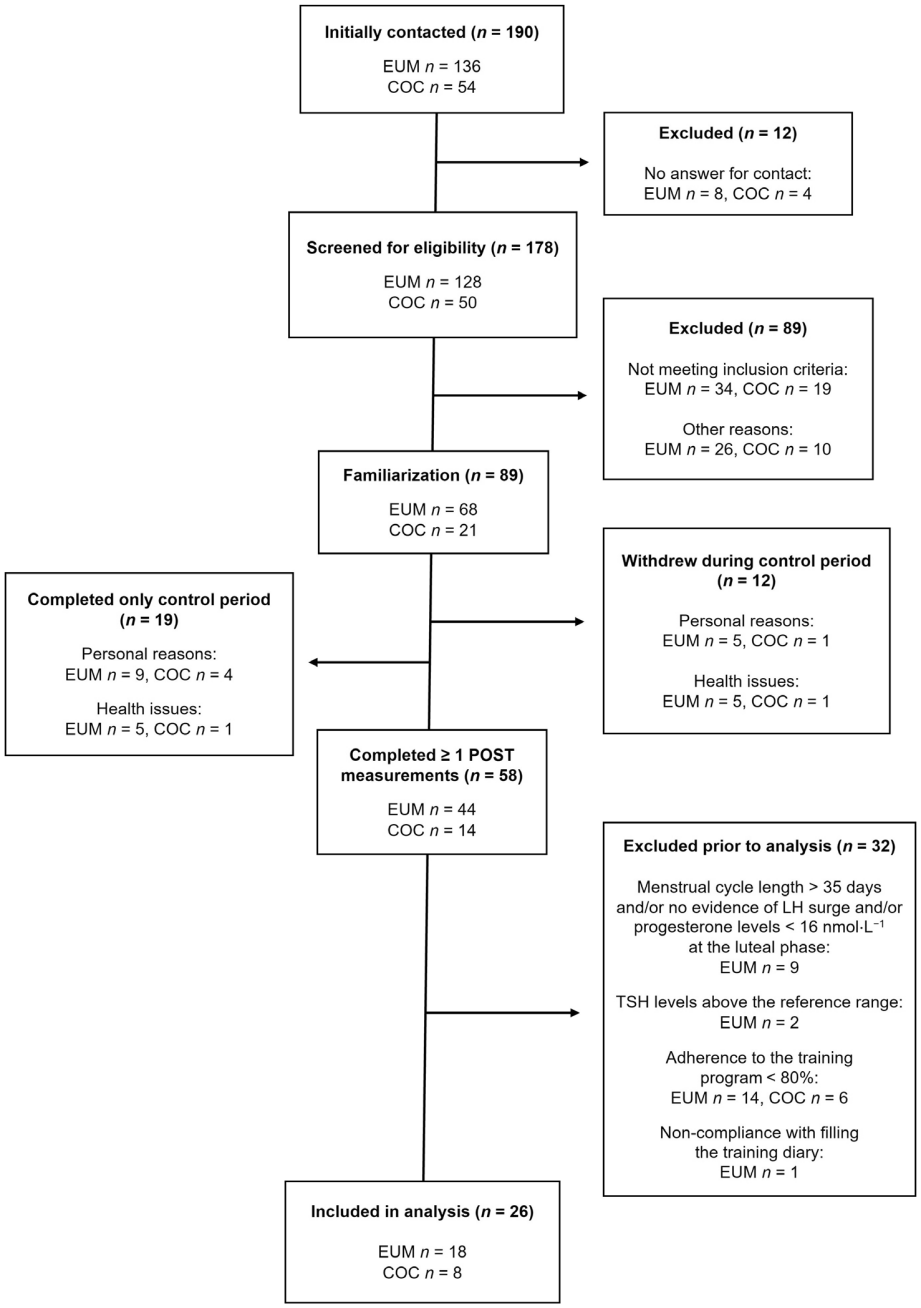
Flowchart illustrating the number of eumenorrheic females (EUM) and females using combined oral contraceptives (COC) at each stage of the study. LH, luteinizing hormone; TSH, thyroid stimulating hormone.

Figure 1 provides an overview of the enrollment process for the study. After exclusion, a total of 89 participants proceeded to the familiarization. From those, 31 participants either completed only the control cycle preceding the training intervention or withdrew during control cycle due to personal or health‐related issues. Of the participants who completed at least one post‐intervention measurement, 32 were excluded due to not meeting the criteria for eumenorrhea (*n* = 9), non‐compliance with the training program with adherence <80% (*n* = 20), non‐compliance with filling the training diary (*n* = 1) or thyroid stimulating hormone concentrations above the reference range (*n* = 2). Eumenorrhea was defined according to criteria by Elliott‐Sale et al. (2021): menstrual cycle lengths ≥21 days and ≤35 days, plus evidence of the luteinizing hormone (LH) surge, plus correct hormonal profile i.e. progesterone concentrations >16 nmol·L^−1^ at the luteal phase of the menstrual cycle, plus no hormonal contraceptive (HC) use 3 months prior to recruitment. Participants were classified as eumenorrheic based on the retrospective analysis of progesterone in the control cycle. One participant was included, in the absence of a positive LH and estrone‐3‐glucuronide (E3G) surge test, as concentrations of progesterone were >16 nmol·L^−1^ at the luteal phase measurement indicating ovulation had occurred. Finally, 26 participants were included in the analysis: 18 eumenorrheic females (EUM) and 8 females using combined oral contraceptives (COC). Participants in COC had used COC pills for at least 6 months prior to the study. COC preparations included differing amounts of estrogenic (0.02–0.03 mg ethinyl estradiol) and third or fourth generation progestin (0.075–3.0 mg) containing pills. Seven participants had regimen of 21 hormone containing pills (active phase) followed by seven hormone‐free days for withdrawal bleed (inactive phase), while one participant had 24 days lasting active phase followed by 4 days inactive phase (Table S1).

### 2.2 Study design and menstrual cycle/hormonal contraceptive phase definitions

The study began with a control cycle, lasting one full menstrual or COC cycle prior to the training intervention. Participants visited the laboratory nine times: once to receive a full familiarization session, and the remaining eight visits for testing (four visits in a fasted state for blood sampling and body composition measurements, and four visits under fed conditions for performance tests). Before the control cycle started, participants were individually familiarized with all equipment and protocols. Participants tested the treadmill by both walking and running on it, practiced countermovement jumps, and performed contractions on the leg press according to the instructions provided by the researcher. All familiarization sessions were conducted by the same two researchers. Participants were given the necessary equipment, including a heart rate monitor with a chest strap at the familiarization session. In addition, an LH and E3G surge detection kit was provided to participants in EUM to monitor morning urine samples. Participants were instructed to use the equipment and fill in training diaries (described below). All participants started menstrual/COC cycle tracking on the first day of the menstrual cycle in the EUM group or on the first day of the active phase i.e. pill‐taking phase in the COC group by filling menstrual/contraceptive diaries daily.

**FIGURE 2:**
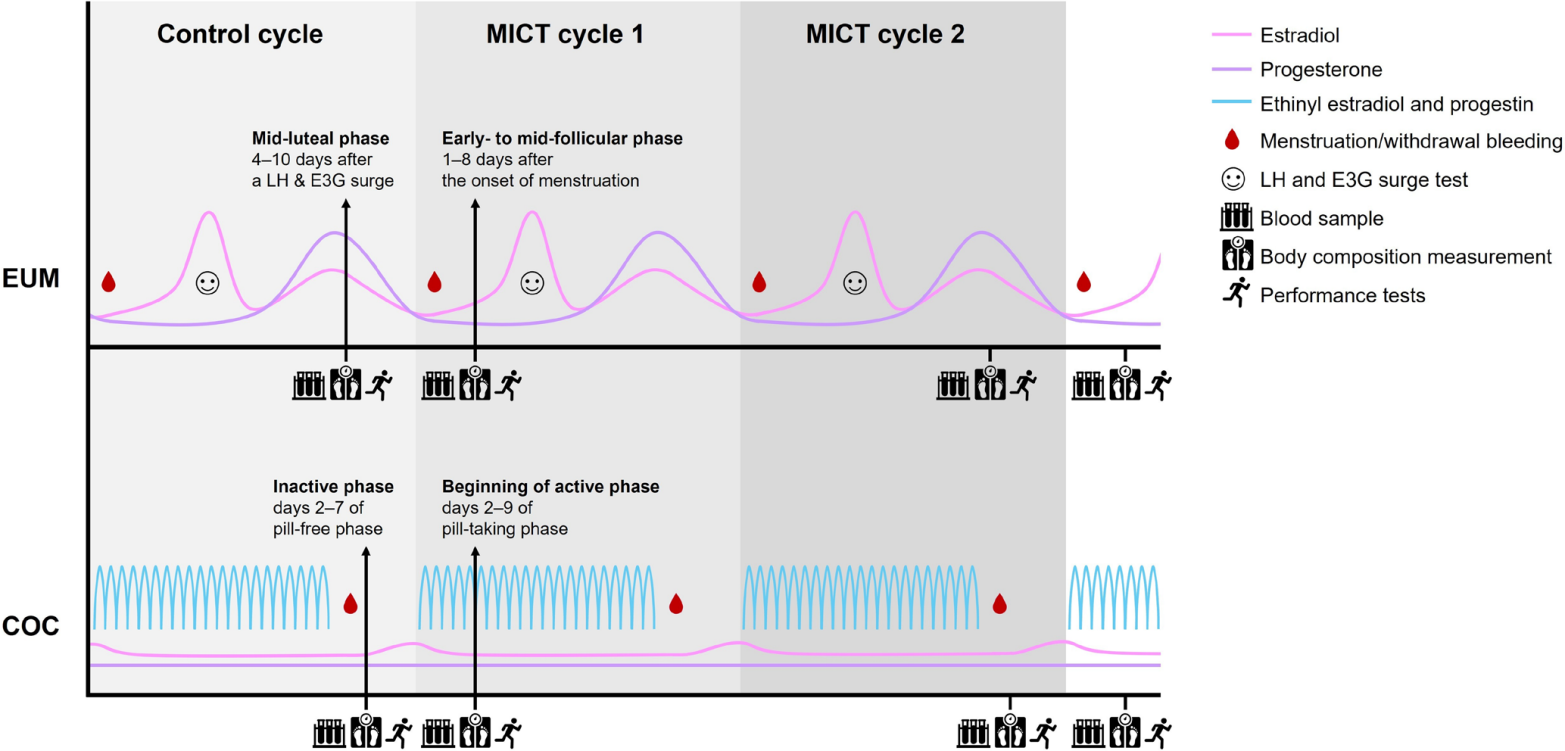
Study design and measurement schedule aligned with expected hormonal fluctuations across the menstrual cycle in eumenorrheic females (EUM) and across combined oral contraceptive pill cycle (COC). Measurements included blood sampling and assessment of body composition and performance tests on two consecutive days in two different phases of the menstrual cycle and COC pill cycle before and after the moderate‐intensity continuous training (MICT) intervention. E3G, estrone‐3‐glucuronide; LH, luteinizing hormone.

Laboratory measurements were conducted on two consecutive days in two different phases of the menstrual cycle and COC pill use before (pre) and after (post) the training intervention. Blood samples were collected and body composition measured after at least 10 h of overnight fasting on the first measurement day. Participants were instructed to abstain 48 h from alcohol and 24 h from strenuous exercise before measurements, but they were allowed to drink a glass of water after waking up. Performance tests were conducted under fed conditions on the second measurement day, followed by three‐day food diaries. Participants were instructed to abstain from caffeine for 4 h prior to arriving for performance assessments. In EUM, the baseline measurements started in the mid‐luteal phase of the control menstrual cycle, 4–10 days after the LH and E3G surge, and were followed by measurements in the early‐ to mid‐follicular phase of the subsequent menstrual cycle, 1–8 days following the onset of bleeding, according to methodological recommendations by Elliott‐Sale et al. (2021). In COC, the baseline measurements were scheduled between days 2 and 7 of the inactive phase (i.e., pill‐free or placebo phase) at the end of the control cycle, and between days 2 and 9 of the active phase (i.e., pill‐taking phase) of the subsequent COC cycle, which initiated the training intervention. Post‐intervention measurements took place at the same phases described above. Figure 2 provides an overview of the study design.

### 2.3 Training intervention

After the control cycle ended, participants started the MICT program (Table 1), which lasted two menstrual or COC cycles (~8 weeks). Training included running or brisk walking at an intensity between 60% and 75% of HR_max_, which was determined in the incremental treadmill running test (described below). The training intervention consisted of 30–90 min long sessions completed three times per week, except during the first and last weeks, when physical performance tests were performed and adequate rest was required prior to tests. This resulted in 615 min scheduled training in total in the first 4‐week cycle and 630 min in the second 4‐week cycle. All sessions included an additional 5‐min warm‐up by walking. The scheduled training prescription was three times per week; however, to encourage adherence to the training program, participants were allowed to move training sessions by a day or two if needed to accommodate work, study, and/or family schedules. As a result, the completed training frequency may have varied between participants. The goal was to maintain similar training volume across participants every 4 weeks. The training prescription aligned with established aerobic physical activity guidelines. The intensity (60%–75% HR_max_) corresponds to moderate‐intensity exercise, and the weekly training volume (3 sessions of 30–90 min) meets or exceeds recommendations by the World Health Organization guideline of >150 min of moderate‐intensity aerobic physical activity per week (Bull et al., 2020). Running was chosen because it is easily accessible with low skill demands and the intensity is easily standardized across fitness levels by adjusting speed.

**TABLE 1:** Moderate‐intensity continuous training (MICT) program over two menstrual/COC cycles.

Week	Duration (min)	Intensity (%HR_max_)
1	30	60–65
	60	60–65
2	45	60–65
	45	70–75
	60	60–65
3	45	60–65
	60	70–75
	75	60–65
4	60	60–65
	60	70–75
	75	60–65
5	45	60–65
	45	70–75
	75	60–65
6	45	60–65
	60	70–75
	90	60–65
7	60	60–65
	60	70–75
	90	60–65
8	60	70–75

*Note*: On weeks 1 and 8, peak fat oxidation and V̇O_2peak_ tests were considered in training volume.

Training sessions were unsupervised due to the restrictions regarding the ongoing COVID‐19 pandemic during the intervention. Each training session was recorded using a heart rate monitor (Garmin® Venu Series, Garmin Ltd., Taiwan) with a chest strap (Garmin® HRM‐Dual, Garmin Ltd., Taiwan) to control the desired training intensity. The training volume and intensity were progressively increased. Details of the sessions were filled in training diaries by participants. Completing at least 80% of the scheduled training volume was set as an acceptable training adherence, which was calculated as the percentage of completed training minutes relative to the minutes scheduled in the training program. Participants were instructed to perform the training sessions at the same time of day to minimize the influence of circadian variation (Teo et al., 2011). Furthermore, participants were instructed to maintain their usual daily physical activity and dietary habits during the study.

### 2.4 Blood samples

Blood samples were collected from the antecubital vein into serum tubes (2 × 6 mL Vacuette, Greiner Bio‐One GmBH, Kremsmünster, Austria) for hormone analyses between 06.00 and 10.00 in the morning. Serum tubes were held 15 min at room temperature before centrifuging them for 10 min at 2245 × g (Megafuge, 1.0R, Heraeus, Germany). After the serum was separated, it was frozen first at −20°C and later stored at −80°C until the final analysis. Concentrations of estradiol and progesterone were measured for validation of menstrual phase. Estradiol (catalog number L2KE22), progesterone (cat. no. L2KPW2), total testosterone (cat. no. L2KTW2), androstenedione (cat. no. L2KAO2), DHEA‐S (cat. no. L2KDS2), and SHBG (cat. no. L2KSH2) were analyzed from serum using chemical luminescence techniques by Immulite®2000 XPi‐analyzator (Siemens Healthcare Diagnostics, New York, USA). Free testosterone (Human Testosterone, Free ELISA, Biovendor, cat. no. RCD016R), DHT [Dihydrotestosterone (DHT) ELISA, Demeditec, cat. no. DE5761], and DHEA (DHEA ELISA, Demeditec, cat. no. DEH3344) were analyzed using enzyme‐linked immunosorbent assay (ELISA) with Dynex DS2®‐analyzator (Dynex Technologies, Chantilly, VA, USA). The inter‐assay coefficients of variation (CV) determined in our laboratory, and analytical sensitivity were 8.7% and 55.0 pmol·L^−1^ for estradiol, 15.5% and 0.3 nmol·L^−1^ for progesterone, 6.7% and 0.5 nmol·L^−1^ for total testosterone, 12.4% and 0.06 pmol·L^−1^ for free testosterone, 14.8% and 5.94 pg.·mL^−1^ for DHT, 11.2% and 1.0 nmol·L^−1^ for androstenedione, 4.7% and 0.28 nmol·L^−1^ for DHEA, 7.3% and 0.08 μmol·L^−1^ for DHEA‐S, and 5.5% and 0.02 nmol·L^−1^ for SHBG.

### 2.5 LH and E3G surge test

Clearblue dual hormone ovulation kits [10‐pack (EAN 4015600566968) and 20‐pack (EAN 4015600566920) Clearblue® Advanced Digital Ovulation Test, SDP Swiss Precision Diagnostics GmbH (SDP), Geneva, Switzerland] were used to monitor urinary surges of LH and E3G (metabolite of estradiol). Participants completed testing at home according to the manufacturer's instructions. Results were used to assess menstrual status and determine measurement schedules.

### 2.6 Anthropometrics and body composition

At the first study visit, each participant's height was measured with a wall‐mounted stadiometer. In the morning, after at least 10 h of overnight fasting, body composition and body mass were assessed using a multifrequency bioelectrical impedance device (Inbody 770, Biospace Co. Ltd., Seoul, Korea). Participants wore underwear and had visited the bathroom before measurements. BMI was calculated as body mass (kg) divided by height squared (m^2^). To avoid the potential influence on eating and physical activity behaviors, participants were not given feedback regarding their body composition results until all measurement sessions were completed.

### 2.7 Incremental treadmill running test

Incremental treadmill running test was performed before and after the MICT intervention on the second measurement day. With the exception of a peak fat oxidation test (walking on treadmill with increasing gradient) completed the day before performance tests on the first measurement day, participants were instructed to refrain from strenuous exercise for the 24 h prior to performance testing. To assess the endurance performance level of the participants, peak oxygen uptake (V̇O_2peak_) was measured on a treadmill (OJK‐1, Telineyhtymä, Kotka, Finland) after strength tests. The test was performed using a standard incremental protocol described in detail elsewhere (Salmi et al., 2025). Oxygen consumption (V̇O_2_) was measured breath‐by‐breath using a gas analyzer calibrated according to manufacturer instructions before every test (Vyntus CPX, Vyaire Medical GmbH, Hoechberg, Germany). V̇O_2peak_ was defined as the highest rolling average 60‐s V̇O_2_ value using K‐lab 3.1.25 software (2121.10.01 Aino Health Management Oy, Helsinki, Finland).

### 2.8 Countermovement jump and isometric leg press

Countermovement jump and isometric leg press were performed before incremental treadmill running test on the second measurement day. Counter movement jump height (CMJ) was measured by flight time with photocells (Faculty of Sport and Health Sciences, University of Jyväskylä, Finland) from a minimum of three maximal effort jumps with 60‐s rest between attempts until the result no longer improved. The jump with the highest jump height was selected for statistical analyses. Maximal isometric force production (*F*_max_) of the leg extensor muscles was measured with isometric horizontal bilateral leg press (Faculty of Sport and Health Sciences, University of Jyväskylä, Finland) from a minimum of three maximal contractions performed with 120‐s rest between attempts until maximal force was obtained. Warm‐up session, measurements and the determination of CMJ and maximal isometric force are described in detail elsewhere (Salmi et al., 2025).

### 2.9 Training and food diaries

Participants reported their training sessions and daily physical activity in Excel template‐based training diaries including date, sport, type of training, duration, distance, rated perceived exertion of session and the description of training. Researchers evaluated the training diaries weekly, and total adherence to training was calculated at the end of the training intervention. Any inconsistencies such as session durations were checked from the Garmin training files using the Golden Cheetah open‐source software (Version 3.6). Energy intake and energy availability were assessed with food and training diaries. Participants recorded their food intake for 3 days, starting the day after laboratory measurements. Participants were instructed to record all foods, drinks, and supplements consumed as grams, estimated as household measures or recorded as photographs as accurately as possible. Furthermore, participants were instructed to maintain their typical diet and refrain from attempting weight loss during the study. The food diaries were analyzed using the Finnish Food Database Fineli (National Institute for Health and Welfare, Helsinki, Finland). Energy availability was calculated by subtracting exercise energy expenditure (EEE) of intentional training sessions or physical activity from energy intake divided by FFM. EEE was calculated using estimated metabolic equivalent of task (MET) values and measured resting energy expenditure as described elsewhere in detail (Löfberg et al., 2025).

### 2.10 Statistical analysis

Data were analyzed using IBM SPSS 30.0.0.0 (IBM Corp., Chicago, IL, USA) and figures were prepared using GraphPad Prism 9.5.1 (GraphPad Software Inc., San Diego, CA, USA) and Microsoft Powerpoint version 2511 (Microsoft Corporation, Redmond, WA, USA). Descriptive data are presented as means with standard deviations for normally distributed variables or medians with interquartile range for non‐normally distributed variables. Normality was evaluated by visual inspection of histograms and Q‐Q plots.

Given the small sample size and the similarity of baseline characteristics in the EUM and COC groups, the groups were pooled together for the analysis in order to increase statistical power while adjusting for the potential confounding effect of group. All models included two separate phase‐specific analyses: (1) the inactive pill phase was aligned with the follicular phase due to similar hormonal environments (low concentrations of estradiol and progesterone) between phases, and (2) the active pill phase was aligned with the luteal phase, while acknowledging that the hormonal environments differ between the two groups at these time points (Elliott‐Sale et al., 2021). The dataset contained several missing values, primarily due to seasonal illness among the participants. As linear mixed models are robust to missing data (Newans et al., 2022), we chose not to exclude these cases in order to preserve the sample size. Details on missing data are provided in Table S2.

Within‐group differences between follicular and luteal phases, between inactive and active phases, and between follicular/inactive and luteal/active phases were examined with paired‐samples Student's *t*‐test for normally distributed variables and Wilcoxon signed‐rank test for non‐normally distributed variables. Changes over the MICT intervention and differences between the groups were assessed with linear mixed‐effects models including group, time, and the group–time interaction as fixed effects. Estradiol and DHEA at follicular/inactive phases, and progesterone, free testosterone, DHT, and DHEA at luteal/active phases were log‐transformed for these analyses. For outcomes that showed statistically significant changes, both the outcome and continuous explanatory variables were standardized in separate models to obtain standardized estimates (*β*).

To test whether the baseline androgen concentrations were associated with the changes in body composition and physical performance over the MICT, we used a multiple linear regression model: post‐intervention body composition or performance was used as the outcome, baseline androgen concentration as the explanatory variable, and the corresponding baseline outcome value as a covariate. To investigate associations between potential changes in androgen concentrations and the changes in body composition and physical performance, we conducted a multiple regression analysis using change scores. If there were statistically significant associations, models were further adjusted for outcome baseline values.

As body composition could confound or mediate the hormone–outcome associations, the following adjustments were applied. To isolate associations with relative adiposity, FFM was added as a covariate to the FM model. Performance‐outcome models were run first unadjusted and then adjusted for body composition. Specifically, both FFM and FM were added as covariates when CMJ was the outcome, whereas *F*_max_ and absolute V̇O_2peak_ models included FFM only. Post‐intervention body composition variables were used for adjusting when investigating associations of pre‐intervention baseline androgen concentrations with changes in outcomes, while change scores of body composition variables were used when investigating associations of changes in androgen concentrations with changes in outcomes. The model assumptions were verified before accepting the results and statistical significance was set at *p* < 0.05.

Because the hormone variables included some relatively high observations in two participants from EUM and one participant from COC that could skew the results, sensitivity analyses were performed after excluding those values that exceeded both 1.5 × interquartile range (Walfish, 2006; Yang et al., 2019) and an absolute *Z*‐score >3. However, no physiological or measurement‐related reasons for these observations were found. Excluded values of estradiol (Hackney et al., 2022; Korad et al., 2022; Soedirdjo et al., 2023), free testosterone (Bui et al., 2015; Huang et al., 2022; Kumar et al., 2005; Søeborg et al., 2014; van der Vange et al., 1990), and SHBG (Wiegratz et al., 2003) were considered physiologically plausible, although most of the previously reported corresponding free testosterone values have been reported in participants with PCOS. Hormone values excluded for sensitivity analyses (three free testosterone values, two DHT and DHEA values, and one estradiol and SHBG value) are presented in Table S3.

## 3 RESULTS

### 3.1 Study participants

EUM and COC groups were largely similar in baseline characteristics (Table 2), except that participants in EUM were slightly older than in COC. Concentrations of estradiol were lower, whereas SHBG concentrations were higher in the COC group compared to EUM at both the follicular/inactive phase and the luteal/active phase (Table 3). At the luteal/active phase progesterone concentrations were higher among EUM. Furthermore, concentrations of DHT were 1.02 nmol·L^−1^ [95% confidence interval (CI) = 0.05–1.99] higher among EUM than in COC in follicular/inactive phase over both time points (*β* = 0.75, *p* = 0.040) in the sensitivity analysis. No within‐group differences were observed in body composition or physical performance outcomes between the follicular and luteal phases, or between the inactive and active phases, at baseline or post‐intervention. In addition, no between‐group differences were detected between the aligned phases (follicular/inactive and luteal/active) at either baseline or post‐intervention for any body composition or physical performance outcomes.

**TABLE 2:** Baseline characteristics of eumenorrheic females (EUM) at follicular phase and females using combined oral contraceptives (COC) at inactive phase.

Group	EUM (*n* = 15–18^b^)	COC (*n* = 7–8^c^)	*p* ^a^
Age (years)	31 ± 4	26 ± 5	0.022
Height (m)	1.67 ± 0.06	1.69 ± 0.05	0.318
Body mass (kg)	67.0 ± 8.3	69.4 ± 7.2	0.501
BMI (kg·m^−2^)	24.2 ± 3.2	24.2 ± 1.7	0.998
Body fat percentage (%)	28.1 ± 6.3	28.3 ± 5.5	0.935
V̇O_2peak_ (mL·kg^−1^·min^−1^)	37.6 ± 4.9	38.6 ± 4.4	0.655
Energy intake (kcal)	2094 ± 343	2037 ± 282	0.689
Energy availability (kcal·kg FFM^−1^·d^−1^)	39.5 ± 6.5	39.5 ± 6.2	0.992
Length of MC or COC phases (days)	28 ± 2	21–24 active +4–7 inactive	
Day of ovulation	14 ± 2		

*Note*: Values are presented as mean ± standard deviation.

Abbreviations: BMI, body mass index; COC, combined oral contraceptive; FFM, fat‐free mass; MC, menstrual cycle; V̇O_2peak_, peak oxygen uptake.

^a^ The *p* values are from independent‐samples Student's *t*‐test.

^b^ For body mass, BMI and body fat percentage *n* = 17, for energy intake and energy availability, *n* = 16, for V̇O_2peak_, *n* = 15.

^c^ For V̇O_2peak_ and energy availability *n* = 7.

**TABLE 3:** Serum hormone concentrations before (pre) and after (post) the MICT intervention across menstrual cycle phases in eumenorrheic females and across pill phases in females using combined oral contraceptives.

	Eumenorrheic females			Females using COC			Pooled sample		
	Pre follicular phase (3)	Post follicular phase (3)	Time	Pre inactive phase (3.5)	Post inactive phase (3)	Time	Group	Time	Group × time
*Hormones*	(*n* = 17)	(*n* = 13)		(*n* = 8)	(*n* = 7)				
Estradiol (pmol·L^−1^)	114.0 (91.3–161.0)	99.1 (78.1–133.5)	0.695	38.6 (24.9–151.3)**	65.3 (32.6–125.0)	0.333	**0.004**	0.571	0.312
Progesterone (nmol·L^−1^)	1.12 (0.70–1.58)	1.26 (0.88–1.69)	0.518	0.72 (0.36–1.21)	0.72 (0.43–1.35)	0.600	0.077	0.423	0.962
Total testosterone (nmol·L^−1^)	0.582 (0.482–0.871)	0.565 (0.351–1.040)	0.748	0.522 (0.240–1.409)	0.496 (0.468–1.270)	0.337	0.444	0.553	0.336
Free testosterone (pmol·L^−1^)	8.508 (4.810–11.446)	8.910 (6.790–13.757)	0.287	4.237 (2.716–7.440)	3.675 (2.254–7.229)	0.918	0.234	0.473	0.580
DHT (nmol·L^−1^)	2.35 (1.45–3.13)	2.89 (1.59–3.26)	0.986	1.54 (0.66–2.29)	1.14 (0.73–1.60)*	0.466	0.052	0.548	0.561
Androstenedione (nmol·L^−1^)	7.49 (4.35–10.55)	8.79 (4.15–10.50)	0.959	5.95 (3.75–12.91)	5.46 (4.52–10.40)	0.664	0.717	0.748	0.703
DHEA (nmol·L^−1^)	51.55 (26.99–74.55)	46.08 (28.15–86.76)	0.691	32.80 (30.25–60.89)	39.00 (28.50–100.44)	0.195	0.757	0.201	0.407
DHEA‐S (μmol·L^−1^)	4.59 (3.76–6.41)	4.72 (3.62–7.53)	0.473	3.81 (1.63–7.48)	2.01 (1.80–5.13)	0.203	0.261	0.533	0.151
SHBG (nmol·L^−1^)	64.7 (49.7–82.4)	50.3 (44.4–83.5)	0.988	234.0 (199.8–281.3)***	257.0 (188.0–270.0)***	0.226	**<0.001**	0.329	0.321

	Pre luteal phase (6)	Post luteal phase (6)	Time	Pre active phase (4)	Post active phase (3)	Time	Group	Time	Group × time
*Hormones*	(*n* = 18)	(*n* = 16–17^a^)		(*n* = 8)	(*n* = 7)				
Estradiol (pmol·L^−1^)	505.0 (410.0–747.0)^###¤¤¤^	485.0 (341.5–583.5)^###¤¤¤^	0.323	60.6 (26.8–194.3)***	53.6 (35.1–136.0)***	0.664	**<0.001**	0.367	0.854
Progesterone (nmol·L^−1^)	30.70 (21.23–35.00)^###¤¤¤^	26.20 (22.20–29.35)^###¤¤¤^	0.455	0.94 (0.69–1.11)***	0.99 (0.81–1.36)***	0.228	**<0.001**	0.540	0.160
Total testosterone (nmol·L^−1^)	0.572 (0.383–1.175)	0.645 (0.399–1.056)	0.747	0.829 (0.617–1.173)	0.631 (0.252–1.620)	0.764	0.412	0.938	0.669
Free testosterone (pmol·L^−1^)	8.355 (3.652–12.308)	8.681 (3.606–13.173)	0.630	6.124 (3.618–11.072)	3.744 (2.999–10.543)	0.138	0.527	0.314	0.133
DHT (nmol·L^−1^)	2.30 (1.55–2.91)	2.01 (1.44–2.86)	0.231	1.50 (0.78–2.46)	1.66 (1.07–2.47)^#^	0.566	0.253	0.848	0.256
Androstenedione (nmol·L^−1^)	8.74 (5.38–11.75)^¤^	8.22 (5.17–13.05)^¤^	0.402	9.26 (5.53–15.38)^#^	7.70 (6.83–19.90)	0.384	0.329	0.239	0.779
DHEA (nmol·L^−1^)	34.44 (25.56–78.62)	41.01 (28.50–96.11)	0.489	42.18 (25.92–72.78)	41.47 (32.69–81.58)	0.215	0.988	0.160	0.495
DHEA‐S (μmol·L^−1^)	5.17 (4.17–6.85)^#¤¤^	5.43 (2.87–5.84)	0.061	4.47 (2.48–7.56)	2.77 (2.32–7.73)^#^	0.849	0.713	0.231	0.374
SHBG (nmol·L^−1^)	70.8 (53.8–86.8)^#^	69.9 (53.4–89.7)	0.925	152.5 (127.0–196.3)***^#^	152.0 (119.0–245.0)***^#^	0.409	**<0.001**	0.518	0.455

*Note*: Values are presented as median and interquartile range (25th–75th percentile). Numbers following the phases indicate the mean measurement day, expressed as a menstrual cycle day (follicular phase) or as days after a positive luteinizing hormone and estrone‐3‐glucuronide test (luteal phase) in EUM, or as a pill‐free day (inactive phase) or a pill day (active phase) in COC. *P* values are from linear mixed‐effects models with random intercepts for participants. *p < 0.05, **p < 0.01, ***p < 0.001 for between‐group differences between pre follicular and pre inactive phases, pre luteal and pre active phases, post follicular and post inactive phases, and post luteal and post active phases. ^#^ *p* < 0.05, ^##^ *p* < 0.01, ^###^ *p* < 0.001 for within‐group differences between pre follicular and pre luteal phases, post follicular and post luteal phases, pre inactive and pre active phases, and post inactive and active phases. ^¤^ *p* < 0.05, ^¤¤^ *p* < 0.01, ^¤¤¤^ *p* < 0.001 for within‐group differences in pooled sample between pre follicular/inactive and pre luteal/active phases, and post follicular/inactive and post luteal/active phases. Significant associations are in bold.

Abbreviations: DHT, dihydrotestosterone; DHEA, dehydroepiandrosterone; DHEA‐S, dehydroepiandrosterone sulfate; SHBG, sex hormone‐binding globulin.

^a^ For DHT, *n* = 16.

### 3.2 Adherence to the intervention

Among included participants, training adherence rate over the two menstrual/COC cycles of MICT was 94% ± 8% in the EUM group and 95% ± 6% in the COC group, being similar between the groups, with a mean difference of 1.2% (95% CI = −8.0%–5.5%, *p* = 0.712). Participants in EUM completed 1147 ± 106 min of training and participants in COC 1154 ± 89 min of training from the planned training program (Table 1).

### 3.3 Training‐induced changes in androgen concentrations

Across the pooled sample, i.e., both groups, no statistically significant changes in androgen concentrations were observed in the follicular/inactive phase or luteal/active phase after the intervention (Table 3). In sensitivity analysis, however, DHEA concentrations increased from baseline to post‐intervention in the luteal/active phase (*p* = 0.039).

### 3.4 Training‐induced changes in body composition and physical performance

**FIGURE 3:**
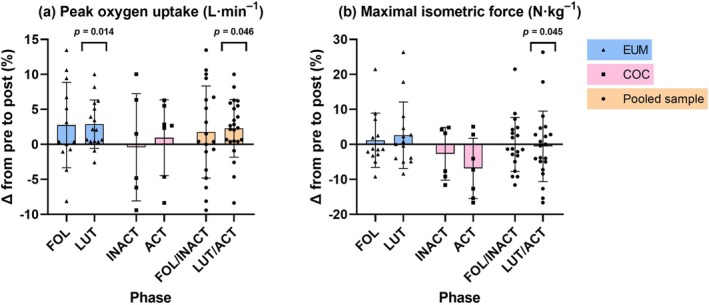
Phase‐specific changes in (a) peak oxygen uptake and (b) maximal isometric force from baseline (pre) to post‐intervention (post) measurements in eumenorrheic females (EUM) at follicular phase (*n* = 13) and luteal phase (a, *n* = 16; b, *n* = 14), in combined oral contraceptive users at inactive phase (*n* = 6) and active phase (*n* = 7), and in the pooled sample at follicular/inactive phases (*n* = 19) and luteal/active phases (a, *n* = 23; b, *n* = 21). Colored bars represent group means and whiskers standard deviations in EUM (blue), COC (pink), and pooled sample (orange), with black symbols representing individual values. *P* values are main effects of time (a), and a group–time interaction term (b) from linear mixed‐effects models.

Across the pooled sample, and within both groups, no statistically significant changes in body composition outcomes were observed after the intervention (Table 4). No main effects of group, time, or interaction were observed for energy intake or energy availability in either of the phases. In the pooled sample, V̇O_2peak_ increased by 0.05 L·min^−1^ (95% CI = 0.001–0.098, *β* = 0.16, *p* = 0.046) in the luteal/active phase (Figure 3a). The within‐group results showed that V̇O_2peak_ increased in the EUM group (*B* = 0.07, 95% CI = 0.02–0.12, *β* = 0.22, *p* = 0.014) but not in the COC group (*B* = 0.03, 95% CI = −0.05–0.11, *β* = 0.10, *p* = 0.447). Adjusting for FFM eliminated the significant time effect in the pooled sample (*B* = 0.05, *p* = 0.097), and in the EUM group (*B* = 0.06, *p* = 0.050).

**TABLE 4:** Body composition and physical performance measures before (pre) and after (post) the MICT intervention across menstrual cycle phases in eumenorrheic females and across pill phases in females using combined oral contraceptives.

	Eumenorrheic females			Females using COC			Pooled sample		
	Pre follicular phase	Post follicular phase	Time	Pre inactive phase	Post inactive phase	Time	Group	Time	Group × time
Body composition	(*n* = 17)	(*n* = 14)		(*n* = 8)	(*n* = 7)				
Fat‐free mass (kg)	47.8 ± 4.3	47.9 ± 4.8	0.960	49.7 ± 6.6	50.9 ± 6.6	0.110	0.328	0.178	0.196
Fat mass (kg)	19.2 ± 6.2	19.1 ± 6.8	0.744	19.7 ± 4.3	19.6 ± 4.2	0.165	0.938	0.187	0.336
Physical performance	(*n* = 15)	(*n* = 13–14)		(*n* = 7)	(*n* = 7)				
Maximal isometric force (N)	2444 ± 616	2569 ± 728	0.700	2540 ± 1028	2380 ± 930	0.241	0.885	0.443	0.237
Counter movement jump height (cm)	23.2 ± 4.6	22.6 ± 4.5	0.516	25.3 ± 3.7	23.6 ± 3.3	0.081	0.350	0.075	0.274
V̇O_2peak_ (L·min^−1^)	2.50 ± 0.26	2.64 ± 0.26	0.099	2.76 ± 0.29	2.72 ± 0.32	0.802	0.133	0.450	0.248

	Pre luteal phase	Post luteal phase	Time	Pre active phase	Post active phase	Time	Group	Time	Group × time
Body composition	(*n* = 18)	(*n* = 17)		(*n* = 8)	(*n* = 7)				
Fat‐free mass (kg)	47.8 ± 4.1	47.9 ± 4.3	0.990	49.9 ± 6.2	49.6 ± 7.3	0.886	0.328	0.910	0.899
Fat mass (kg)	19.0 ± 6.3	18.9 ± 6.3	0.230	19.2 ± 4.7	18.9 ± 4.4	0.726	0.889	0.344	0.716
Physical performance	(*n* = 17–18)	(*n* = 15–16)		(*n* = 8)	(*n* = 7)				
Maximal isometric force (N)	2568 ± 588	2684 ± 643	0.246	2598 ± 1021	2455 ± 1118	0.091	0.964	0.456	**0.045**
Counter movement jump height (cm)	24.0 ± 4.7	24.0 ± 3.3	0.098	24.1 ± 3.4	24.1 ± 3.7	0.742	0.737	0.490	0.224
V̇O_2peak_ (L·min^−1^)	2.53 ± 0.30	2.59 ± 0.30	**0.014**	2.71 ± 0.30	2.74 ± 0.39	0.447	0.246	**0.046**	0.418

*Note*: Values are presented as mean ± standard deviation. *P* values are from linear mixed‐effects models with random intercepts for participants. Significant associations are in bold.

Abbreviation: V̇O_2peak_, peak oxygen uptake.

While *F*_max_ did not change significantly in the pooled sample or within‐groups, group–time interaction terms identified a significant interaction for unadjusted *F*_max_ (*B* = −218.83, 95% CI = −432.43 to −5.22, *β* = −0.29, *p* = 0.045); however, this was observed only in the luteal/active phase (Table 4 and Figure 3b), indicating that the change in *F*_max_ measured in the luteal/active phase might differ between the groups. However, the within‐group estimates showed that the change in *F*_max_ was not significant in either group (*B* = 70.55, *β* = 0.09, *p* = 0.246 for EUM, and *B* = −148.28, *β* = −0.20, *p* = 0.091 for COC). Adjusting for FFM eliminated this significant interaction effect (*B* = −204.97, *β* = −0.27, *p* = 0.070). Furthermore, a significant group–time interaction was found for FFM‐adjusted *F*_max_ in the follicular/inactive phase (*B* = −194.43, 95% CI = −383.54 to −5.33, *β* = −0.26, *p* = 0.045). Within‐group estimates indicated that FFM‐adjusted *F*_max_ decreased in the COC group (*B* = −169.48, 95% CI = −326.52 to −12.43, *β* = −0.22, *p* = 0.036), while the change was non‐significant in the EUM group (*B* = 24.96, 95% CI = −80.56–130.48, *β* = 0.03, *p* = 0.623).

### 3.5 Associations between androgen concentrations and changes in body composition

Based on the multiple regression analysis, pre‐intervention androgen and SHBG concentrations, as well as changes in androgen and SHBG concentrations from baseline to post‐intervention, were not associated with training‐induced changes in body composition outcomes either in the follicular/inactive phase or luteal/active phase (for associations between changes, see Table S4).

### 3.6 Associations between androgen concentrations and changes in physical performance

**FIGURE 4:**
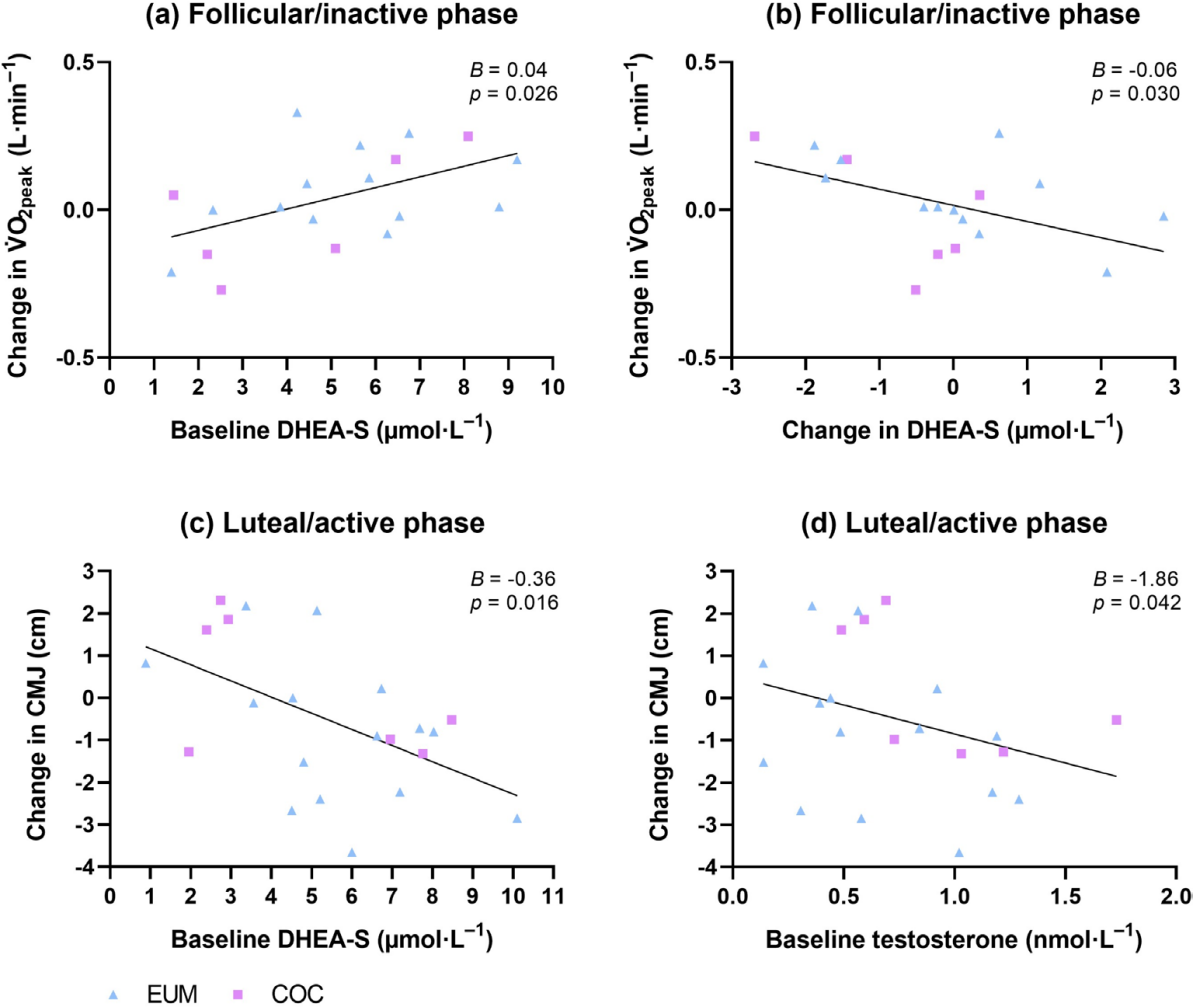
Associations between (a) baseline (pre‐intervention) dehydroepiandrosterone sulfate (DHEA‐S) concentrations and changes in peak oxygen uptake (V̇O_2peak_) (*n* = 19), (b) changes in DHEA‐S concentrations and changes in V̇O_2peak_ (*n* = 18), (c) baseline DHEA‐S concentrations and changes in counter movement jump height (CMJ) (*n* = 22), and (d) baseline testosterone concentrations and changes in CMJ (*n* = 22). Unstandardized coefficients (*B*) and *p* values are from multiple linear regression analyses using baseline and post‐intervention outcome values (a, c, d) and change scores (b).

Across the pooled sample, a significant positive association was observed between baseline DHEA‐S concentrations and change in FFM‐adjusted V̇O_2peak_ in follicular/inactive phase (*B* = 0.04, 95% CI = 0.01–0.07, *β* = 0.31, *p* = 0.026). Furthermore, a significant negative association was observed between baseline testosterone concentrations and change in CMJ (*B* = −1.86, 95% CI = −3.65 to −0.07, *β* = −0.23, *p* = 0.042) and between baseline DHEA‐S concentrations and change in CMJ (*B* = −0.36, 95% CI = −0.64 to −0.08, *β* = −0.25, *p* = 0.016) in the luteal/active phase. Adjusting for post‐intervention FFM and FM eliminated these significant associations (*B* = −1.44, 95% CI = −3.27 to 0.39, *β* = −0.18, *p* = 0.114 for testosterone, *B* = −0.27, 95% CI = −0.58 to 0.04, *β* = −0.19, *p* = 0.087 for DHEA‐S). In Figure 4a,c,d associations are illustrated between baseline androgen concentrations and change scores of outcomes.

A multiple regression analysis revealed a significant negative association between change scores in DHEA‐S and in V̇O_2peak_ only in the follicular/inactive phase (Table 5 and Figure 4b). This association persisted after adjusting for change in FFM (*B* = −0.06, 95% CI = −0.106 to −0.006, *β* = −0.54, *p* = 0.030) and after controlling for baseline V̇O_2peak_. Excluding extreme hormone values in the sensitivity analyses did not materially alter results regarding analyses investigating associations.

**TABLE 5:** Associations of changes in androgen and SHBG concentrations with changes in physical performance.

	Follicular/inactive phase								
	Counter movement jump height (CMJ)			Maximal isometric force production (*F*_max_)			Aerobic capacity (V̇O_2peak_)		
	*B* (95% CI)	*β*	*p*	*B* (95% CI)	*β*	*p*	*B* (95% CI)	*β*	*p*
Total testosterone	0.277 (−3.497, 4.050)	0.041	0.877	146.830 (−125.136, 418.796)	0.282	0.268	0.050 (−0.237, 0.338)	0.096	0.715
Free testosterone	−0.040 (−0.372, 0.292)	−0.068	0.799	20.914 (−3.126, 45.955)	0.426	0.083	−0.011 (−0.038, 0.015)	−0.222	0.389
DHT	0.469 (−0.629, 1.567)	0.231	0.375	16.570 (−72.074, 105.214)	0.101	0.696	0.002 (−0.089, 0.093)	0.011	0.966
Androstenedione	−0.029 (−0.360, 0.302)	−0.048	0.854	6.949 (−19.421, 33.320)	0.140	0.583	−0.021 (−0.045, 0.004)	−0.411	0.096
DHEA	0.009 (−0.005, 0.023)	0.335	0.201	0.324 (−0.850, 1.499)	0.150	0.565	−0.001 (−0.002, 0.000)	−0.317	0.220
DHEA‐S	−0.305 (−1.117, 0.507)	−0.211	0.434	11.306 (−47.240, 69.853)	0.109	0.686	−0.058 (−0.108, −0.007)	−0.547	**0.030**
SHBG	0.000 (−0.036, 0.036)	−0.004	0.989	1.682 (−1.043, 4.407)	0.323	0.208	−9.110×10^−5^ (−0.003, 0.003)	−0.017	0.948

	Luteal/active phase								
	Counter movement jump height (CMJ)			Maximal isometric force production (*F*_max_)			Aerobic capacity (V̇O_2peak_)		
	*B* (95% CI)	*β*	*p*	*B* (95% CI)	*β*	*p*	*B* (95% CI)	*β*	*p*
Total testosterone	0.262 (−1.789, 2.313)	0.059	0.792	42.291 (−224.799, 309.782)	0.072	0.742	−0.089 (−0.205, 0.027)	−0.336	0.124
Free testosterone	0.080 (−0.153, 0.313)	0.159	0.483	−4.052 (−35.584, 25.479)	−0.076	0.732	−0.002 (−0.016, 0.013)	−0.054	0.812
DHT	0.104 (−0.898, 1.106)	0.048	0.830	77.664 (−46.959, 202.288)	0.267	0.206	−0.033 (−0.093, 0.026)	−0.255	0.257
Androstenedione	−0.129 (−0.358, 0.100)	−0.249	0.254	7.252 (−24.257, 38.761)	0.102	0.635	−0.008 (−0.022, 0.005)	−0.272	0.213
DHEA	0.001 (−0.012, 0.014)	0.032	0.885	0.571 (−1.123, 2.265)	0.150	0.488	0.000 (−0.001, 0.000)	−0.260	0.240
DHEA‐S	0.259 (−0.528, 1.046)	0.151	0.499	−15.203 (−132.319, 101.913)	−0.058	0.788	0.007 (−0.041, 0.055)	0.064	0.776
SHBG	−0.020 (−0.072, 0.031)	−0.185	0.415	1.996 (−4.670, 8.662)	0.135	0.537	−0.001 (−0.004, 0.002)	−0.208	0.356

*Note*: Unstandardized coefficients (*B*), standardized coefficients (*β*), confidence intervals (CI), and *p* values are from a multiple linear regression analysis. Significant associations are in bold.

Abbreviations: DHT, dihydrotestosterone; DHEA, dehydroepiandrosterone; DHEA‐S, dehydroepiandrosterone sulfate; SHBG, sex hormone‐binding globulin.

## 4 DISCUSSION

The main finding was the absence of statistically significant changes in androgen concentrations after the intervention, while V̇O_2peak_ increased in the pooled sample and in eumenorrheic females (EUM) in the luteal/active phase. However, sensitivity analysis showed that DHEA concentrations increased from baseline to post‐intervention. Baseline DHEA‐S concentrations were positively associated with the change in FFM‐adjusted V̇O_2peak_ in the follicular/inactive phase. Furthermore, a negative association was observed between the change in DHEA‐S concentrations and the change in V̇O_2peak_ over the MICT intervention in the follicular/inactive phase.

### 4.1 Changes in androgen concentrations, body composition and physical performance

While endurance training‐related decreases in basal estradiol concentrations have been reported in follicular phase of regularly menstruating females with a history of informal running experience (Boyden et al., 1983), and in luteal phase of untrained females (Keizer et al., 1987), Smith et al. (2011) reported no changes in estradiol, testosterone, or SHBG concentrations in sedentary eumenorrheic premenopausal females after training 150 min per week (5 times a week for 30 min) of moderate‐intensity aerobic exercise for four menstrual cycles (14–18 weeks), consistent with our findings. Also, reports of the changes in androgen concentrations due to chronic physical exercise have been inconsistent in females (Enea et al., 2011). While some studies have found no significant difference in basal plasma DHEA‐S concentrations after a 6‐month endurance training period (Ronkainen et al., 1986), others have reported a decrease in DHEA‐S concentrations at the follicular and luteal phases after a 3‐month endurance training program (Keizer et al., 1987), as well as after 18–20 months of endurance training in previously untrained females (Keizer et al., 1989). This could be related to a decrease in adrenocorticotrophic hormone (ACTH) after endurance training, which can reflect diminished adrenal drive (Keizer et al., 1987). Recently, Hackney et al. (2024) found that DHEA‐S concentrations were suppressed after treadmill running after 24 h of recovery in the follicular phase, and these reductions in DHEA‐S concentrations were correlated with increases in cortisol concentrations at volitional fatigue and 90 min of recovery. ACTH, released from the pituitary gland, stimulates the adrenal glands to secrete glucocorticoids and androgens, such as cortisol and DHEA‐S (Miller & Auchus, 2011). Therefore, changes in DHEA‐S concentrations may reflect hypothalamic–pituitary–adrenal axis activity. In the present study, however, DHEA‐S concentrations remained stable over MICT.

As expected, there were no significant changes in FFM over MICT, even though lean mass (Smith et al., 2011) and muscle size may increase after endurance training in untrained individuals (Konopka & Harber, 2014). Fat mass (FM) remained statistically unchanged following the MICT intervention, which may be due to the relatively short duration of the intervention and the absence of dietary control. These results support some existing findings—for example, in a study by Carter et al. (2001), 7 weeks of moderate‐intensity endurance exercise training led to no changes in FFM, FM, or basal serum estradiol and testosterone concentrations in premenopausal females. Although running‐based interventions have often resulted in reductions in body mass and fat percentage, these effects are typically observed in programs lasting over a year (Hespanhol Junior et al., 2015). In the present study, the average weekly training volume of approximately 144 min may have been insufficient to produce measurable changes in body composition. Furthermore, changes in body composition depend not only on energy expenditure but also on energy intake. The balance between these factors determines whether conditions are favorable for changes in FM and FFM. Previous studies have shown that endurance training can reduce FM even without dietary changes (Skrypnik et al., 2015; Willis et al., 2012). However, exercise alone typically results in only modest weight loss compared to interventions that combine exercise with dietary modifications (Shaw et al., 2006). In contrast, combining endurance training with strength training may be a more effective stimulus for inducing changes in body composition, as it leads to greater muscle hypertrophy than endurance training alone (Wilson et al., 2012).

It is worth noting that significant performance adaptations were found only in one menstrual cycle/COC phase. The increase in V̇O_2peak_ was observed in the pooled sample only in the luteal/active phase and in the EUM group in the luteal phase. However, the magnitude of these improvements was small. Clinically meaningful improvement in cardiorespiratory fitness is often pragmatically defined as an increase of ~3.5 mL·kg^−1^·min^−1^ (1 MET) in body weight–scaled V̇O_2peak_ (Ross et al., 2016), although this threshold is not universally established and is influenced by changes in body mass. In our cohort, the observed increases were smaller (0.9 mL·kg^−1^·min^−1^, *p* = 0.010 for pooled sample; 1.1 mL·kg^−1^·min^−1^, *p* = 0.005 for EUM), suggesting limited clinical relevance. While McNulty et al. (2020) suggested in their meta‐analysis that exercise performance of eumenorrheic females might be trivially reduced during the early follicular phase of the menstrual cycle, compared to other phases, this does not offer a physiological explanation for the different adaptations in V̇O_2peak_ observed across the phases after MICT intervention. It could be speculated if the impairing effect of the early follicular phase could confound the slight improvements observed in V̇O_2peak_. Furthermore, adjusting for FFM eliminated the significant change in V̇O_2peak_, indicating that changes in FFM partially accounted for the changes.

The change in unadjusted *F*_max_ in the luteal/active phase and FFM‐adjusted *F*_max_ in the follicular/inactive phase appeared to differ depending on COC use. The change in *F*_max_, adjusted for FFM, was non‐significant in the EUM group in the follicular phase, while a significant decrease was observed in the COC group in the inactive phase. Although COCs have a suppressing effect on endogenous production of androgens (Haverinen et al., 2022; Kangasniemi et al., 2022; Zimmerman et al., 2014), this decrease cannot be attributed to androgens, as no significant changes in androgen concentrations were observed after MICT in the COC group. The differences between groups might be explained by FFM, as results differed with FFM‐adjustment. However, no significant differences in FFM were observed between COC and EUM at baseline or at post‐intervention.

The androgenicity of the used COC pills may have influenced the maintenance of muscle strength during the endurance training program, as androgenicity of COCs has been suggested to impact performance (Ruzić et al., 2003). Ruzić et al. (2003) found that females using androgenic COCs (levonorgestrel) had a greater increase in FFM and muscle strength in response to a strength training program compared to females using anti‐androgenic COCs. In a study by Ihalainen et al. (2019), the authors found that females using HC had smaller gains in lean mass compared to those who had never used HC in response to combined strength and endurance training. Furthermore, FM decreased significantly in females not using HC, while the decrease in females using HC was non‐significant (Ihalainen et al., 2019). In our study, androgenicity of COCs varied between the participants. Six of the participants were using COCs that included anti‐androgenic progestin (dienogest or drospirenone) while three of the participants used formulations including androgenic progestins (desogestrel or gestodene). Thus, the possible effect of the androgenicity of HC on responses to exercise should be considered when interpreting results from studies including HCs with differing androgenicity.

### 4.2 Associations between androgen concentrations and changes in body composition and physical performance

Baseline androgen and SHBG concentrations and changes in androgen and SHBG concentrations from baseline to post‐intervention were not significantly associated with body composition. In terms of performance outcomes, a significant positive association was observed between baseline DHEA‐S concentrations and change in FFM‐adjusted V̇O_2peak_, while a significant negative association was observed between changes in DHEA‐S concentrations and changes in V̇O_2peak_, regardless of whether the change in FFM was included in the model. These findings indicate that greater increases in FFM‐adjusted V̇O_2peak_ were observed with higher baseline DHEA‐S concentrations, and greater reductions in DHEA‐S concentrations from baseline to post‐intervention were associated with greater increases in V̇O_2peak_, vice versa. The magnitude of these associations, traditionally considered small for baseline DHEA‐S and moderate for changes in DHEA‐S, indicated that baseline DHEA‐S concentrations were associated with changes in aerobic capacity to a small extent, whereas longitudinal shifts in DHEA‐S concentrations over time were moderately and inversely related to aerobic capacity adaptations. However, these associations were observed only in the follicular/inactive phase, where the DHEA‐S concentrations were lower than in the luteal/active phase at baseline. These observations are further supported by the negative association between the DHEA‐S concentrations and V̇O_2peak_ found in our previous cross‐sectional study (Salmi et al., 2025) suggesting that high concentrations of DHEA‐S may represent poorer aerobic capacity. On the other hand, resting serum DHEA‐S concentration has previously been suggested to be a potential anabolic hormone marker of adaptation to resistance training among young females (Aizawa et al., 2003). Aizawa et al. (2003) found that resting serum DHEA‐S concentrations increased after a resistance training period, and the change in DHEA‐S concentrations was positively correlated with the change in lean body mass. It could be speculated if changes in DHEA‐S concentrations can reflect the type of training and adaptations achieved; however, future studies are necessary to support or refute this supposition.

A small but significant negative association was found between baseline testosterone concentrations and change in counter movement jump height (CMJ) and between baseline DHEA‐S concentrations and change in CMJ in the luteal/active phase. The research on associations between androgen concentrations and jump/power performance in healthy females is limited. In previous cross‐sectional studies, higher total and free testosterone concentrations have been associated with enhanced jump performance (Cardinale & Stone, 2006) and power performance (Bermon & Garnier, 2017). Further, the free androgen index has been positively associated with resistance training‐induced changes in muscle mass and strength in normally menstruating females and females using HC (Alexander et al., 2024), and with resistance training‐induced changes in muscle cross‐sectional area in pre‐menopausal females (Häkkinen et al., 1992). In both studies, total testosterone concentrations did not correlate with the changes in muscle force or mass. However, it is worth noting that ∼15% of the participants in the study by Alexander et al. (2024) were diagnosed with PCOS. In the present study, adjusting for post‐intervention FFM and FM eliminated significant associations between CMJ and baseline testosterone concentrations and DHEA‐S concentrations, suggesting that body composition potentially accounted for these associations.

### 4.3 Strengths and limitations

A strength of our study is controlling menstrual cycle and COC pill phases and conducting measurements in both follicular/inactive and luteal/active phases, as different phases of the cycle might influence both androgen concentrations (Braunstein et al., 2011; Bui et al., 2013; Goebelsmann et al., 1974; Rothman et al., 2011; Salonia et al., 2008) and performance variables (McNulty et al., 2020). Furthermore, all models were adjusted for the use of hormonal contraceptives, as the use of COCs decreases concentrations of androgens (Haverinen et al., 2022; Kangasniemi et al., 2022; Salmi et al., 2026; Zimmerman et al., 2014), thus being a potential confounding factor in the associations between androgen concentrations and body composition and performance outcomes. However, for practical reasons, we did not control dose and type of COC, so dose and androgenicity of COCs varied between participants.

Another limitation that should be considered is the lack of a control group in the MICT intervention, which calls into question if the observed changes and associations were due to the intervention or other factors. Furthermore, the length of the intervention may have been too short and the training volume insufficient to induce marked changes in body composition, which may have limited our ability to detect body composition‐related associations. The sample size was relatively small due to the rigorous nature of tracking menstrual cycles and only including eumenorrheic participants defined according to criteria by Elliott‐Sale et al. (2021). The distribution between eumenorrheic females and females using COC was uneven, which may have limited our ability to detect differences in outcomes. These factors prevented us from investigating whether the use of COC moderated associations.

There are few limitations that should be considered regarding methods for androgen and body composition determination. The used immunoassay method can be considered as a limitation, as mass spectrometry offers higher sensitivity and specificity for investigating low testosterone concentrations, which are expected in females (Jasuja et al., 2022; Kanakis et al., 2019; Stanczyk et al., 2003; Taieb et al., 2003). Furthermore, concerns have been raised regarding the accuracy and reliability of direct immunoassay methods for measuring free testosterone. Determining free testosterone by ELISA may be limited by greater imprecision and suboptimal specificity compared with reference methods such as equilibrium dialysis coupled with liquid chromatography–tandem mass spectrometry (Jasuja et al., 2022; Rosner et al., 2007; van Uytfanghe et al., 2005). Although dual‐energy X‐ray absorptiometry (DXA) is usually considered as the gold standard method for measuring body composition (Achamrah et al., 2018), our study used bioelectrical impedance analysis (BIA), as the research regulations in Finland and the Ethical Committee restrict the frequent use of DXA due to concerns about radiation dose. However, FFM and FM are the most accurate BIA metrics, as they have been shown to correlate strongly with corresponding DXA measures (Achamrah et al., 2018; Potter et al., 2022). Furthermore, the same protocol, including participants wearing light clothing, being in a fasted state, and visiting the bathroom before measurements and measurements being conducted at the same time of day, was followed in every measurement. Considering these limitations and the exploratory nature of this study, findings should be interpreted with caution.

Participants were instructed to follow their habitual eating patterns to avoid any intervention‐induced changes resulting from changes in diet, rather than endurance training. It is important to bear in mind that even though energy balance was unchanged, MICT was not always scheduled during the days when participants filled food and training diaries. This may have affected exercise energy expenditure. Participants completed three‐day food diaries only after the laboratory measurements to avoid testing days to interfere with food diaries. Thus, it is possible that energy availability during the intervention might have been slightly different. Furthermore, it is possible that participants' energy intake fluctuated during the intervention despite instructions to maintain a habitual diet constant. However, the unchanged body composition supports the observation of stable energy availability during the intervention. Participants were likely not at risk for low energy availability, as energy availability was approximately 40 kcal·kg FFM^−1^·d^−1^.

The MICT intervention seemed not to cause such physiological or psychological stress that would disrupt the function of the hypothalamic–pituitary–ovarian axis, as all participants had progesterone concentrations above 16 nmol·L^−1^ at the luteal phase of the menstrual cycle, also at the end of the intervention, indicating ovulation. Further, all participants had menstrual cycle lengths ≥21 days and ≤35 days and evidence of the LH and E3G surge on the second training cycle, except one who had lengthened 43 days long menstrual cycle, which is why the measurement of LH and E3G surge was scheduled incorrectly. Even though she did not have a positive LH and E3G surge test, concentrations of progesterone were >16 nmol·L^−1^ at the luteal phase. It is worth considering that from naturally menstruating females who were enrolled to the study, 20% were excluded retrospectively prior to analysis due to not meeting the criteria for eumenorrhea. However, over a third of menstrual cycles are suggested to be anovulatory in spontaneously menstruating females, without hormonal contraception (Prior et al., 2015), which poses challenges for interpreting our results in a way that reflects the real‐life variability of hormonal profiles among females.

## 5 CONCLUSIONS

Our results showed that ~8‐week MICT did not markedly alter androgen concentrations, but baseline DHEA‐S concentrations and changes in DHEA‐S concentrations were associated with changes in V̇O_2peak_ in healthy, premenopausal females. However, these changes and associations were not consistently observed in both menstrual cycle/combined oral contraceptive phases studied. This might be due to differences in sex steroid concentrations between phases or other factors having a greater influence on training adaptations than androgen concentrations. Thus, DHEA‐S concentrations might represent a marker of endurance training‐induced changes in V̇O_2peak_ rather than being a key determinant of it. Additionally, the results indicate that hormonal contraceptive use might affect how individuals respond to endurance training, as changes in V̇O_2peak_ and F_max_ appeared to vary by contraceptive use status. However, given the sample size and the observational nature of several associations, these results should be interpreted with caution and confirmed in future studies. These findings highlight the limited and inconclusive knowledge regarding the role of androgen concentrations in the endurance training adaptations.

## AUTHOR CONTRIBUTIONS

**Vera M. Salmi:** Conceptualization; data curation; formal analysis; funding acquisition; investigation; methodology; project administration; visualization; writing – original draft; writing – review and editing. **Jari E. Karppinen:** Formal analysis; writing – review and editing. **Maarit Lehti:** Supervision; writing – review and editing. **Heikki Kyröläinen:** Conceptualization; supervision; writing – review and editing. **Johanna K. Ihalainen:** Conceptualization; methodology; resources; supervision; writing – review and editing. **Ritva S. Mikkonen:** Conceptualization; data curation; funding acquisition; methodology; project administration; resources; supervision; writing – original draft; writing – review and editing.

## FUNDING INFORMATION

Women’s menstrual cycle and endurance training study (NaisQs) was funded by the Finnish Ministry of Education and Culture (OKM/21/626/2021, OKM/101/626/2021, OKM/82/626/2022) and Firstbeat Analytics Oy. Garmin Venu 2S smartwatches and Garmin HRM‐dual heart rate monitors were provided by Firstbeat Analytics Oy. Additional funding for blood analyses was provided by Suomen Urheilututkimussäätiö.

## CONFLICT OF INTEREST STATEMENT

The authors declare that they have no conflict of interest.

## ETHICS STATEMENT

The research was approved by the Ethical Committee of the University of Jyväskylä (1519/13.00.04/2021).

## Supporting information


**Table S1:** Hormone components and brand names of combined oral contraceptives.

**Table S2:** Hormone values exceeding both 1.5 × interquartile range and an absolute *Z*‐score >3 in eumenorrheic females (EUM) and in females using combined oral contraceptives (COC).

**Table S3:** Summary of missing data and reasons across all measurements in eumenorrheic females (EUM) and in females using combined oral contraceptives (COC).

**Table S4:** Associations between changes in androgen and SHBG concentrations and changes in body composition.

## Data Availability

The data presented in this study are available from the corresponding author on reasonable request.
