# Supplementary material for: Associations between androgen levels and endurance training‐induced changes in body composition and physical performance in premenopausal females
Source: Physiol Rep. 2026 Apr 14;14(7):e70857. doi: 10.14814/phy2.70857 (PMC13079422; doi:10.14814/phy2.70857)
Supplement: Supplementary file 1 — Table S1: Hormone components and brand names of combined oral contraceptives. [file PHY2-14-e70857-s004.docx]

**Table S1.** Hormone components and brand names of combined oral contraceptives.

|  | **Pills included (*n*)** | **Content (mg/mg)** | **Brand names (duration of active phase + inactive phase)** |
| --- | --- | --- | --- |
| ***Third generation pills*** | |  |  |
|  | Ethinyl estradiol coupled with gestodene (1) | 0.03/0.075 | Gestinyl (21 + 7 days) |
|  | Ethinyl estradiol coupled with desogestrel (1) | 0.02/0.15 | Daisynelle (21 + 7 days) |
| ***Fourth generation pills*** | |  |  |
|  | Ethinyl estradiol coupled with drospirenone (1) | 0.02/3 | Stefaminelle (24 + 4 days) |
|  | Ethinyl estradiol coupled with drospirenone (2) | 0.03/3 | Tasminetta, and Yasmin (21 + 7 days) |
|  | Ethinyl estradiol coupled with dienogest (3) | 0.03/2 | Dienorette (21 + 7 days) |
