# Supplementary material for: Associations between androgen levels and endurance training‐induced changes in body composition and physical performance in premenopausal females
Source: Physiol Rep. 2026 Apr 14;14(7):e70857. doi: 10.14814/phy2.70857 (PMC13079422; doi:10.14814/phy2.70857)
Supplement: Supplementary file 2 — Table S2: Hormone values exceeding both 1.5 × interquartile range and an absolute Z‐score >3 in eumenorrheic females (EUM) and in females using combined oral contraceptives (COC). [file PHY2-14-e70857-s002.docx]

**Table S2.** Summary of missing data and reasons across all measurements in eumenorrheic females (EUM) and in females using combined oral contraceptives (COC).

| **Variable** | **Follicular/inactive phase** | | **Luteal/active phase** | |
| --- | --- | --- | --- | --- |
|  | **Pre** | **Post** | **Pre** | **Post** |
| **Body mass** | EUM *n* = 1 (illness) | EUM *n* = 3 (wrong phase)  EUM *n* = 1 (scheduling conflict)  COC *n* = 1 (illness) |  | EUM *n* = 1 (illness)  COC *n* = 1 (illness) |
| **Body fat percentage** | EUM *n* = 1 (illness) | EUM *n* = 3 (wrong phase)  EUM *n* = 1 (scheduling conflict)  COC *n* = 1 (illness) |  | EUM *n* = 1 (illness)  COC *n* = 1 (illness) |
| **Energy intake** | EUM *n* = 1 (illness)  EUM *n* = 1 (food diary not returned) | EUM *n* = 3 (wrong phase)  EUM *n* = 1 (scheduling conflict)  EUM *n* = 1 (food diary not returned)  COC *n* = 1 (illness) | EUM *n* = 1 (food diary not returned)  COC *n* = 1 (food diary not returned) | EUM *n* = 2 (illness)  COC *n* = 1 (illness) |
| **Energy availability** | EUM *n* = 1 (illness)  EUM *n* = 1 (food diary not returned)  COC *n* = 1 (food diary not returned) | EUM *n* = 3 (wrong phase)  EUM *n* = 1 (scheduling conflict)  EUM *n* = 1 (food diary not returned)  COC *n* = 1 (illness) | EUM *n* = 1 (food diary not returned)  COC *n* = 1 (food diary not returned) | EUM *n* = 2 (illness)  COC *n* = 1 (illness) |
| **Estradiol** | EUM *n* = 1 (illness) | EUM *n* = 3 (wrong phase)  EUM *n* = 1 (scheduling conflict)  EUM *n* = 1 (unsuccessful blood sampling)  COC *n* = 1 (illness) |  | EUM *n* = 1 (illness)  COC *n* = 1 (illness) |
| **Progesterone** | EUM *n* = 1 (illness) | EUM *n* = 3 (wrong phase)  EUM *n* = 1 (scheduling conflict)  EUM *n* = 1 (unsuccessful blood sampling)  COC *n* = 1 (illness) |  | EUM *n* = 1 (illness)  COC *n* = 1 (illness) |
| **Total testosterone** | EUM *n* = 1 (illness) | EUM *n* = 3 (wrong phase)  EUM *n* = 1 (scheduling conflict)  EUM *n* = 1 (unsuccessful blood sampling)  COC *n* = 1 (illness) |  | EUM *n* = 1 (illness)  COC *n* = 1 (illness) |
| **Free testosterone** | EUM *n* = 1 (illness) | EUM *n* = 3 (wrong phase)  EUM *n* = 1 (scheduling conflict)  EUM *n* = 1 (unsuccessful blood sampling)  COC *n* = 1 (illness) |  | EUM *n* = 1 (illness)  COC *n* = 1 (illness) |
| **DHT** | EUM *n* = 1 (illness) | EUM *n* = 3 (wrong phase)  EUM *n* = 1 (scheduling conflict)  EUM *n* = 1 (unsuccessful blood sampling)  COC *n* = 1 (illness) |  | EUM *n* = 1 (illness)  EUM *n* = 1 (value above measuring range)  COC *n* = 1 (illness) |
| **Androstenedione** | EUM *n* = 1 (illness) | EUM *n* = 3 (wrong phase)  EUM *n* = 1 (scheduling conflict)  EUM *n* = 1 (unsuccessful blood sampling)  COC *n* = 1 (illness) |  | EUM *n* = 1 (illness)  COC *n* = 1 (illness) |
| **DHEA** | EUM *n* = 1 (illness) | EUM *n* = 3 (wrong phase)  EUM *n* = 1 (scheduling conflict)  EUM *n* = 1 (unsuccessful blood sampling)  COC *n* = 1 (illness) |  | EUM *n* = 1 (illness)  COC *n* = 1 (illness) |
| **DHEA-S** | EUM *n* = 1 (illness) | EUM *n* = 3 (wrong phase)  EUM *n* = 1 (scheduling conflict)  EUM *n* = 1 (unsuccessful blood sampling)  COC *n* = 1 (illness) |  | EUM *n* = 1 (illness)  COC *n* = 1 (illness) |
| **SHBG** | EUM *n* = 1 (illness) | EUM *n* = 3 (wrong phase)  EUM *n* = 1 (scheduling conflict)  EUM *n* = 1 (unsuccessful blood sampling)  COC *n* = 1 (illness) |  | EUM *n* = 1 (illness)  COC *n* = 1 (illness) |
| **Fat-free mass** | EUM *n* = 1 (illness) | EUM *n* = 3 (wrong phase)  EUM *n* = 1 (scheduling conflict)  COC *n* = 1 (illness) |  | EUM *n* = 1 (illness)  COC *n* = 1 (illness) |
| **Fat mass** | EUM *n* = 1 (illness) | EUM *n* = 3 (wrong phase)  EUM *n* = 1 (scheduling conflict)  COC *n* = 1 (illness) |  | EUM *n* = 1 (illness)  COC *n* = 1 (illness) |
| **Maximal isometric force** | EUM *n* = 2 (illness)  EUM *n* = 1 (wrong phase)  COC *n* = 1 (illness) | EUM *n* = 3 (wrong phase)  EUM *n* = 1 (scheduling conflict)  COC *n* = 1 (illness) | EUM *n* = 1 (data lost due to device being switched off) | EUM *n* = 2 (illness)  EUM *n* = 1 (invalid data file)  COC *n* = 1 (illness) |
| **Counter movement jump height** | EUM *n* = 2 (illness)  EUM *n* = 1 (wrong phase)  COC *n* = 1 (illness) | EUM *n* = 3 (wrong phase)  EUM *n* = 1 (scheduling conflict)  COC *n* = 1 (illness) |  | EUM *n* = 2 (illness)  EUM *n* = 1 (equipment failure)  COC *n* = 1 (illness) |
| **Absolute V̇O_2peak_** | EUM *n* = 2 (illness)  EUM *n* = 1 (wrong phase)  COC *n* = 1 (illness) | EUM *n* = 3 (wrong phase)  EUM *n* = 1 (scheduling conflict)  COC *n* = 1 (illness) |  | EUM *n* = 2 (illness)  COC *n* = 1 (illness) |

DHT, dihydrotestosterone; DHEA, dehydroepiandrosterone; DHEA-S, dehydroepiandrosterone sulfate; pre, baseline measurement; post, post-intervention measurement; SHBG, sex hormone binding globulin; V̇O_2peak_, peak oxygen uptake
