# Supplementary material for: Associations between androgen levels and endurance training‐induced changes in body composition and physical performance in premenopausal females
Source: Physiol Rep. 2026 Apr 14;14(7):e70857. doi: 10.14814/phy2.70857 (PMC13079422; doi:10.14814/phy2.70857)
Supplement: Supplementary file 3 — Table S3: Summary of missing data and reasons across all measurements in eumenorrheic females (EUM) and in females using combined oral contraceptives (COC). [file PHY2-14-e70857-s003.docx]

**Table S3.** Hormone values exceeding both 1.5 × interquartile range and an absolute Z-score > 3 in eumenorrheic females (EUM) and in females using combined oral contraceptives (COC).

|  | **Follicular/inactive phase** | | | | |
| --- | --- | --- | --- | --- | --- |
| Participant | Estradiol  (pmol·L^–1^) | Free testosterone (pmol·L^–1^) | DHT  (nmol·L^–1^) | DHEA  (nmol·L^–1^) | SHBG  (nmol·L^–1^) |
| 1. (EUM) | 353 (pre) |  |  |  |  |
| 2. (EUM)* |  | 37.028 (pre), 31.432 (post) | 7.22 (pre) | 533.40 (pre) |  |
|  | **Luteal/active phase** | | | | |
| Participant | Estradiol  (pmol·L^–1^) | Free testosterone (pmol·L^–1^) | DHT  (nmol·L^–1^) | DHEA  (nmol·L^–1^) | SHBG  (nmol·L^–1^) |
| 2. (EUM)* |  | 29.17 (pre) | 7.08 (pre) | 532.96 (pre) |  |
| 1. (COC) |  |  |  |  | 284 (pre) |

DHT, dihydrotestosterone; DHEA, dehydroepiandrosterone; pre, baseline measurement; post, post-intervention measurement; SHBG, sex hormone binding globulin

*Values are from same participant
