# Supplementary material for: Associations between androgen levels and endurance training‐induced changes in body composition and physical performance in premenopausal females
Source: Physiol Rep. 2026 Apr 14;14(7):e70857. doi: 10.14814/phy2.70857 (PMC13079422; doi:10.14814/phy2.70857)
Supplement: Supplementary file 4 — Table S4: Associations between changes in androgen and SHBG concentrations and changes in body composition. [file PHY2-14-e70857-s001.docx]

**Table S4.** Associations between changes in androgen and SHBG concentrations and changes in body composition.

|  | **Follicular/inactive phase** | | | | | |
| --- | --- | --- | --- | --- | --- | --- |
|  | **Fat-free mass** | | | **Fat mass** | | |
|  | ***B* (95% CI)** | ***β*** | ***p*** | ***B* (95% CI)** | ***β*** | ***p*** |
| **Total testosterone** | −1.106 (−2.851, 0.638) | −0.306 | 0.198 | 0.629 (−1.315, 2.572) | 0.164 | 0.504 |
| **Free testosterone** | −0.035 (−0.221, 0.150) | −0.093 | 0.694 | 0.026 (−0.174, 0.226) | 0.065 | 0.787 |
| **DHT** | −0.211 (−0.829, 0.406) | −0.165 | 0.480 | 0.455 (−0.177, 1.087) | 0.335 | 0.147 |
| **Androstenedione** | −0.094 (−0.268, 0.079) | −0.258 | 0.267 | 0.079 (−0.110, 0.268) | 0.205 | 0.387 |
| **DHEA** | −0.004 (−0.012, 0.004) | −0.246 | 0.298 | 0.003 (−0.005, 0.012) | 0.194 | 0.420 |
| **DHEA-S** | −0.060 (−0.472, 0.353) | −0.073 | 0.764 | 0.014 (−0.430, 0.459) | 0.016 | 0.947 |
| **SHBG** | −0.005 (−0.025, 0.016) | −0.115 | 0.637 | −0.003 (−0.025, 0.018) | −0.077 | 0.754 |
|  | **Luteal/active phase** | | | | | |
|  | **Fat-free mass** | | | **Fat mass** | | |
|  | ***B* (95% CI)** | ***β*** | ***p*** | ***B* (95% CI)** | ***β*** | ***p*** |
| **Total testosterone** | 0.345 (−0.936, 1.626) | 0.122 | 0.581 | 0.151 (−1.451, 1.753) | 0.043 | 0.846 |
| **Free testosterone** | −0.072 (−0.223, 0.078) | −0.218 | 0.329 | 0.096 (−0.091, 0.282) | 0.232 | 0.297 |
| **DHT** | 0.010 (−0.656, 0.675) | 0.007 | 0.976 | 0.112 (−0.701, 0.925) | 0.064 | 0.777 |
| **Androstenedione** | 0.035 (−0.116, 0.186) | 0.105 | 0.634 | −0.050 (−0.237, 0.137) | −0.121 | 0.583 |
| **DHEA** | 0.003 (−0.006, 0.011) | 0.148 | 0.506 | −0.002 (−0.013, 0.009) | −0.078 | 0.725 |
| **DHEA-S** | −0.167 (−0.679, 0.345) | −0.149 | 0.505 | 0.199 (−0.438, 0.835) | 0.142 | 0.523 |
| **SHBG** | −0.006 (−0.039, 0.027) | −0.084 | 0.708 | −0.008 (−0.049, 0.032) | −0.093 | 0.675 |

DHT, dihydrotestosterone; DHEA, dehydroepiandrosterone; DHEA-S, dehydroepiandrosterone sulfate; SHBG, sex hormone binding globulin

Unstandardized coefficients (*B*), standardized coefficients (*β*), confidence intervals (CI), and *p* values are from a multiple linear regression analysis
